# Expression of microtubule-associated protein TPX2 in human gastric carcinoma and its prognostic significance

**DOI:** 10.1186/s12935-016-0357-7

**Published:** 2016-10-10

**Authors:** Cuijie Shao, Changsheng Duan, Jiani Wang, Shunlian Luan, Yong Gao, Dan Jin, Deqiang Wang, Yuming Li, Lihua Xu

**Affiliations:** 1 Department of Pain, Affiliated Hospital of Binzhou Medical College, Binzhou, 256600 China; 2 Department of Gastrointestinal Surgery, Affiliated Hospital of Binzhou Medical College, Binzhou, 256600 China; 3 Breast Cancer Center, The Third Affiliated Hospital of Sun Yat-sen University, Guangzhou, China; 4 Department of Oncology, Affiliated Hospital of Binzhou Medical College, Binzhou, 256600 China; 5 Department of Hematology, The First Affiliated Hospital of Guangzhou Medical University, 1 Kangda Road, Guangzhou, 510230 China; 6 Guangdong Key Laboratory of Urology, The First Affiliated Hospital of Guangzhou Medical University, Guangzhou, Guangdong China

**Keywords:** Gastric carcinoma, TPX2, Prognostic marker

## Abstract

**Background:**

The molecular mechanisms underlying the development and progression of gastric carcinoma remain poorly understood. The main objective of this study was to investigate the expression level of targeting protein for Xenopus kinesin-like protein 2 (TPX2) and its clinical significance in human gastric carcinoma.

**Methods:**

Real-time quantitative polymerase chain reaction (RT-PCR) and western blotting were used to determine the mRNA and protein levels of TPX2 in 20 paired gastric carcinoma tissues and the adjacent normal tissues, and the expression of TPX2 protein in 106 specimens of a gastric carcinoma tissue microarray was determined by immunohistochemistry. The associations of TPX2 expression with the clinicopathological features were analyzed, and the prognosis of gastric carcinoma patients was evaluated.

**Results:**

The results showed that the expression of TPX2 mRNA was significantly higher in gastric carcinoma than in the adjacent normal tissues in 20 paired samples. Western blotting analysis revealed that TPX2 protein was differentially increased in 17 of 20 specimens from primary human gastric carcinoma tissues compared with those from adjacent non-tumor tissues. Immunohistochemical staining showed that TPX2 over-expression was significantly associated with advanced age (P = 0.001) and tumor T stage (P = 0.003). In addition, TPX2 was an independent prognostic factor for overall survival (OS) in the multivariate analysis [hazard ratio (HR) 0.001; 95 % confidence interval (CI) 2.626–7.198; P = 0.001].

**Conclusions:**

TPX2 is up-regulated in gastric carcinoma and is associated with old age and tumor T stage. TPX2 may serve as a good prognostic indicator in patients with gastric carcinoma.

**Electronic supplementary material:**

The online version of this article (doi:10.1186/s12935-016-0357-7) contains supplementary material, which is available to authorized users.

## Background

Among cancers, gastric carcinoma is ranked second worldwide as a cause of mortality [1], with 85,000 people diagnosed with the disease each year [2] and 65,000 people dying of the disease in China each year [3]. Gastric carcinoma is not sensitive to chemotherapy and radiotherapy [4, 5], and its prognosis remains poor [6], primarily because of the lack of a specific prognostic index [7]. Thus, it is of great importance to identify better biomarkers for gastric carcinoma in order to make a prognosis and guide therapeutic options. Targeting protein for Xenopus kinesin-like protein 2 (TPX2) is a microtubule-associated protein that is distributed in the S and G2 phases of the cell cycle and plays an important role in the formation of the mitotic spindle [8, 9]. TPX2 over-expression occurs in various malignant tumors, resulting in abnormal cell centrosome amplification [10], aneuploid cell formation, and malignant transformation [11]. However, no report concerning TPX2 expression and its prognostic value in gastric carcinoma exists in the literature. In this study, we sought to investigate the expression of TPX2 and its associated prognostic significance in gastric carcinoma.

## Methods

### Patients and tissue specimens

The ethics committee at the Binzhou Medical College Hospital approved this study, and all the patients provided written informed consent for the use of clinical specimens for medical research. For real-time polymerase chain reaction (PCR) and Western blot analysis, twenty paired gastric carcinoma and adjacent normal tissues from Binzhou Medical College Hospital between April 2014 and July 2014 were collected. In addition, 106 paraffin-embedded gastric carcinoma specimens were collected between February 2002 and December 2003 for immunohistochemical (IHC) assays. The median age of the patients was 53 years (range 28–75 years), and the median tumor size was 5.6 cm (range 0.5–16 cm, one full stomach infiltration). All the patients were diagnosed with gastric adenocarcinoma. None of them received any type of neoadjuvant therapy, and all underwent curative surgery. Gastrectomy plus standard D2 lymphadenectomy was performed by the same surgical team. After surgery, chemotherapy was administered to patients with advanced-stage disease, but none of the patients received radiation treatment. Tumor stage and differentiation grade were assessed according to the 7th Edition of the UICC/AJCC TNM Staging System. Both tumor and adjacent non-tumor tissues (the adjacent non-tumor tissue was defined as being located at least 5 cm from the tumor edge) were processed immediately after surgery. The initial event in the survival analysis was the surgical treatment time of the patients, and the time of patient death was the end time; this interval was defined as the survival time of patients.

### RT-PCR analysis

Total RNA from human gastric carcinoma tissues and the adjacent normal gastric tissues was extracted using TRIzol reagent (Invitrogen, Carlsbad, CA) according to the manufacturer’s instructions. The RNA was purified using RNeasy Micro columns (Qiagen, Valencia, CA), and 1 μg of RNA from each sample was used for cDNA synthesis primed with random hexamers. Quantitative real-time PCR was then performed using SYBR Green I (TaKaRa, Dalian, CHN) (Applied Biosystems Step-one TM Real-Time PCR System). Each reaction was run in triplicate in three independent experiments. For PCR amplification, TPX2-specific primers were used with denaturation at 95 °C for 10 min, followed by 35 denaturation cycles at 95 °C for 20 s, primer annealing at 58 °C for 20 s, and a primer extension phase at 72 °C for 20 s. A final extension step at 72 °C for 5 min was performed before the reaction mixture was stored at 4 °C. The primer sequences were as follows: *TPX2*: sense 5′-ACCTTGCCCTACTAAGATT-3′, antisense 5′-AATGTGGCACAGGTTGAGC-3′; *GAPDH*: sense 5′-CTCCTCCTGTTCGACAGTCAG-C-3′, antisense 5′-CCCAATACGACCAAATCCGTT-3′. Expression data were normalized to the geometric mean of glyceraldehyde-3-phosphate dehydrogenase (GAPDH) to control the variability in the expression levels. In all the cases, the validity of amplification was confirmed by the presence of a single peak in the melting temperature analysis and linear amplification with an increasing number of PCR cycles.

### Western blot analysis

Fresh gastric carcinoma tissues and normal gastric tissues were ground to a powder in liquid nitrogen and were lysed with sodium dodecyl sulfate-polyacrylamide gel electrophoresis (SDS-PAGE) sample buffer. To detect TPX2 expression at the protein level, equal protein samples (30 μg) extracted from the tissues were separated on 8 % SDS-polyacrylamide gels and transferred to polyvinylidene fluoride (PVDF) membranes (Immobilon P; Millipore, Bedford, MA). The membranes were blocked with 5 % fat-free milk in Tris-buffered saline containing 0.1 % Tween-20 (TBST) for 0.5 h at room temperature. The membranes were incubated with primary antibodies against TPX2 (1:500; Proteintech, USA, 11741-1-AP) for 3 h at room temperature and then with horseradish peroxidase-conjugated goat anti-rabbit IgG (Santa Cruz Biotechnology, SC-2004). Immunoreactive signals for mature TPX2 (100 kDa) were detected using an enhanced chemiluminescent substrate (SuperSignal West Pico Chemiluminescent Substrate; Pierce Chemical, Rockford, IL, USA). GAPDH (1:1000, Proteintech, USA) was used as a loading control.

### Tissue microarray (TMA) preparation

A representative tissue area was first selected on a hematoxylin and eosin (HE)-stained slide for preparation of the TMA sections. Next, the selected area was punched out using a biopsy needle, and a 3-mm tissue core was transferred to a recipient block. Two tissue cores of gastric tumor and an additional normal gastric tissue core were deposited in a paraffin block using a semi-automated tissue arrayer.

### Immunohistochemistry

IHC staining was performed according to the manufacturer’s protocol. The TMA slides were baked for 1 h at 65 °C, deparaffinized with xylene, and rehydrated using a graded ethanol series and distilled water. After the sections were immersed twice in PBS, peroxidase blocking solution was added dropwise to block endogenous peroxidase activity, and the paraffin sections were incubated at room temperature for 10 min. Non-immune bovine serum was added, and the samples were incubated at room temperature for 10 min. The sections were incubated with TPX2 antibodies overnight at 4 °C. The paraffin sections were washed three times with PBS and then incubated with biotin-conjugated anti-rabbit secondary antibody at room temperature for 2 h, followed by incubation in streptavidin–biotin peroxidase solution at room temperature for 10 min and color development with 3,3′-diaminobenzidine (DAB).

The IHC scores were determined by the average score assigned by two pathologists. Immunoreactivities were scored by the intensity of staining (0 no staining; 1 weak = light yellow; 2 moderate = yellow brown; 3 strong = brown) and the percentage of stained cells (0 no staining; 1, 0–10 %; 2, 11–30 %; 3, 31–70 %; 4, >71 %). By multiplication of both values, a final score ranging between 0 and 12 was obtained. TPX2 over-expression was defined as a final score greater than zero.

### Statistical analysis

The TPX2 mRNA transcriptional levels in the tumor and matched non-tumor tissues were compared by the paired sample t test method. The TPX2 positivity rates in tumor and matched adjacent non-tumor tissues were compared using the Chi square test. Clinicopathological features of TPX2-positive patients and TPX2-negative patients were compared using the Chi square test for categorical variables and Student’s t test for continuous data. Overall survival (OS) was defined as the time from surgery until death. Patients who were alive and disease-free were censored at the date of the last follow-up. The Kaplan–Meier method was used to analyze the OS of patients, and comparisons were performed using the log-rank test. Cox’s proportional hazards model was used for multivariate analysis, and the adjusted hazard ratios (HRs) and their 95 % confidence intervals (CIs) were calculated. All the statistical analyses were conducted using the Statistical Software Package for the Social Sciences (SPSS Inc., Chicago, IL, USA). All the statistical tests were two-sided, and a *p* value less than 0.05 was considered statistically significant.

## Results

### TPX2 is up-regulated in gastric carcinoma

**Fig. 1:**
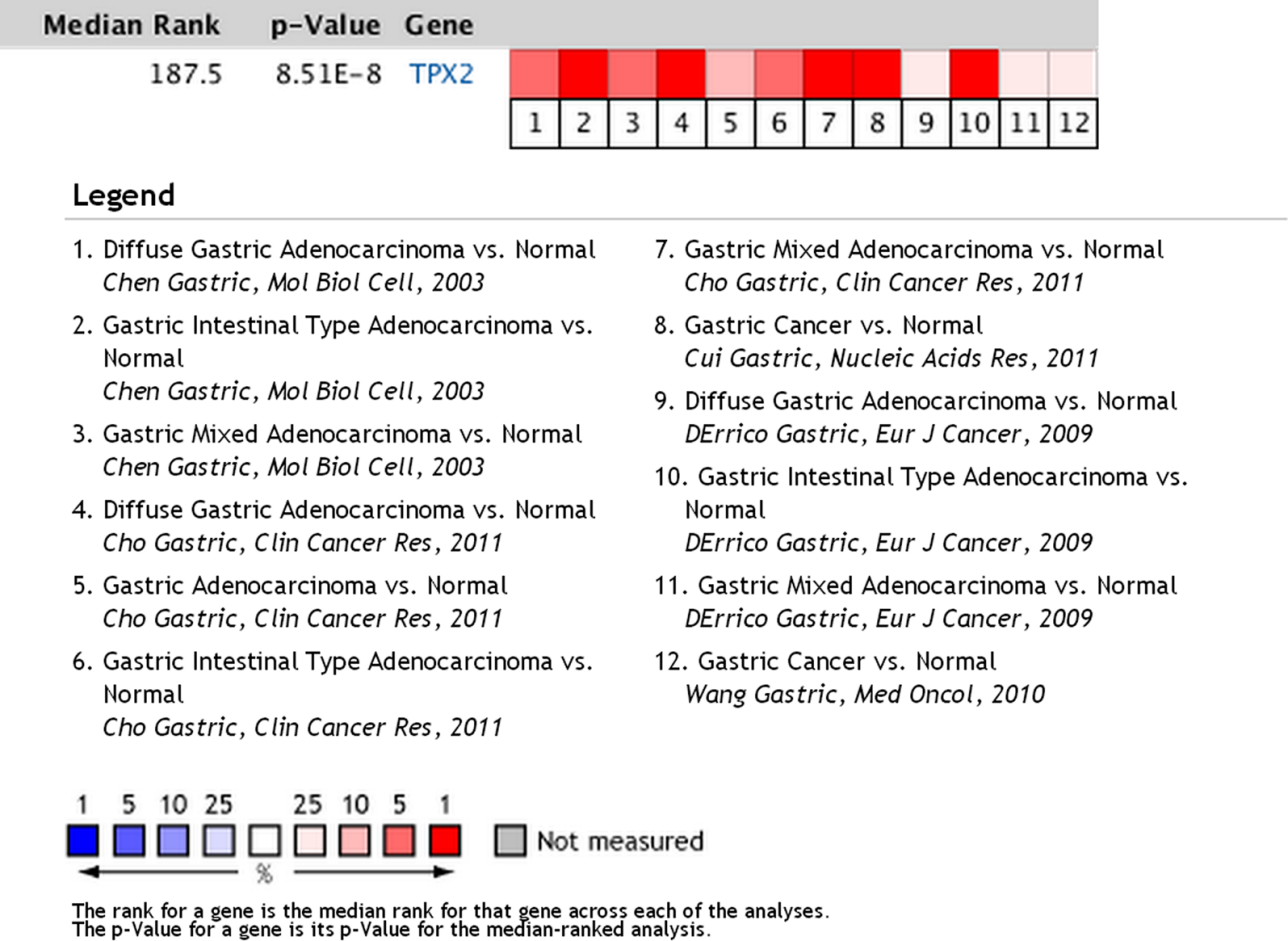
*TPX2* expression is up-regulated in human gastric carcinomas in the Oncomine database. Oncomine heat map of *TPX2* gene expression in clinical gastric carcinoma samples compared with normal gastric tissues (http://www.oncomine.org). In the meta-analysis, *TPX2* expression was significantly higher than that in the corresponding normal tissues in gastric carcinoma, with a median rank of 187.5 and a *p* value of 8.51E−8

**Fig. 2:**
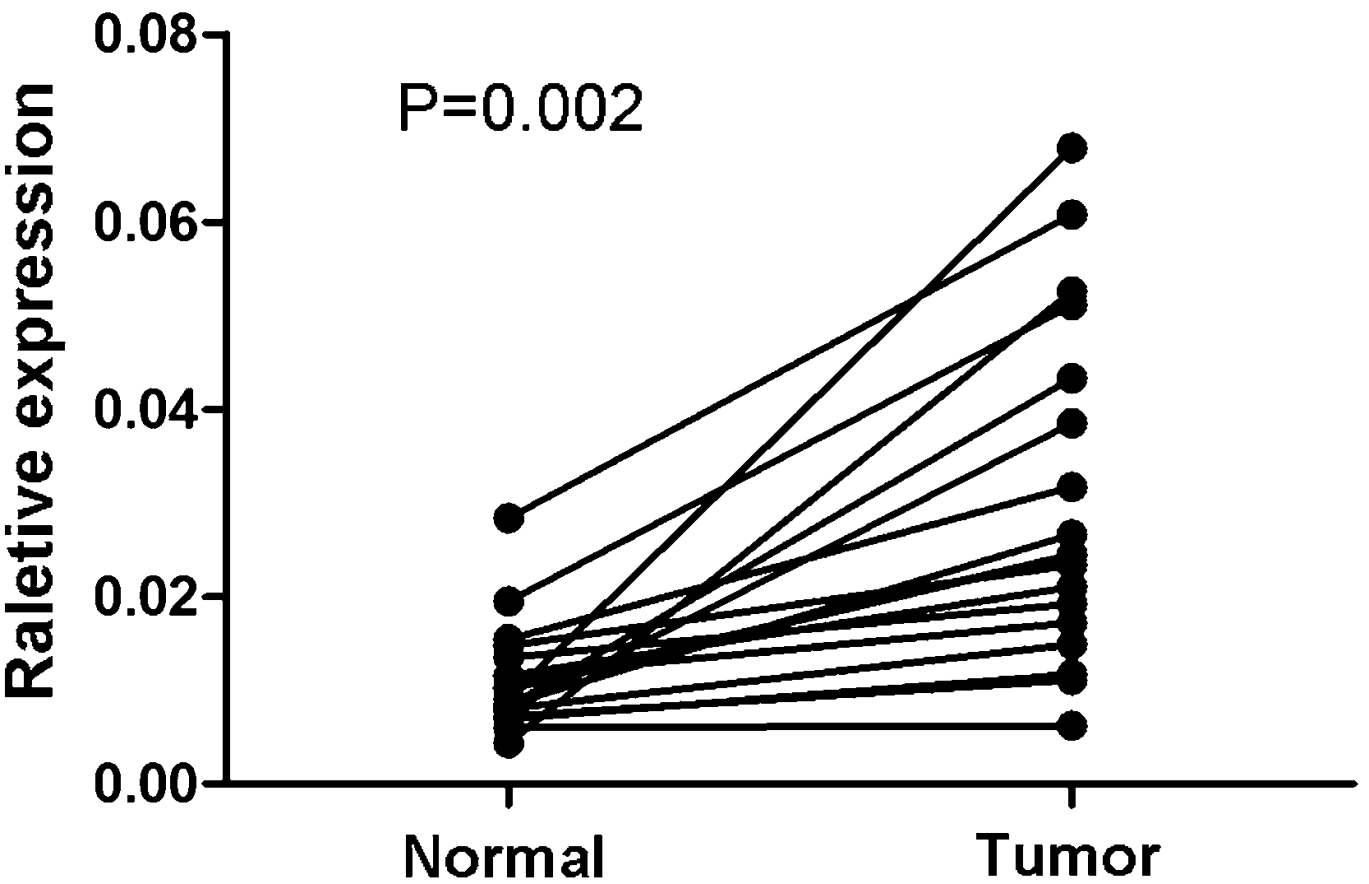
Overexpression of *TPX2* mRNA in gastric carcinoma tissues. Expression levels of *TPX2* mRNA in 20 paired gastric carcinoma tissues according to real-time PCR. The results show that the expression levels of *TPX2* mRNA were significantly higher in tumor tissues than in the paired normal tissues (*P* = 0.002). Normal, matched adjacent non-tumor gastric tissues. Tumor, gastric carcinoma tissues. GAPDH was used as the internal control

**Fig. 3:**
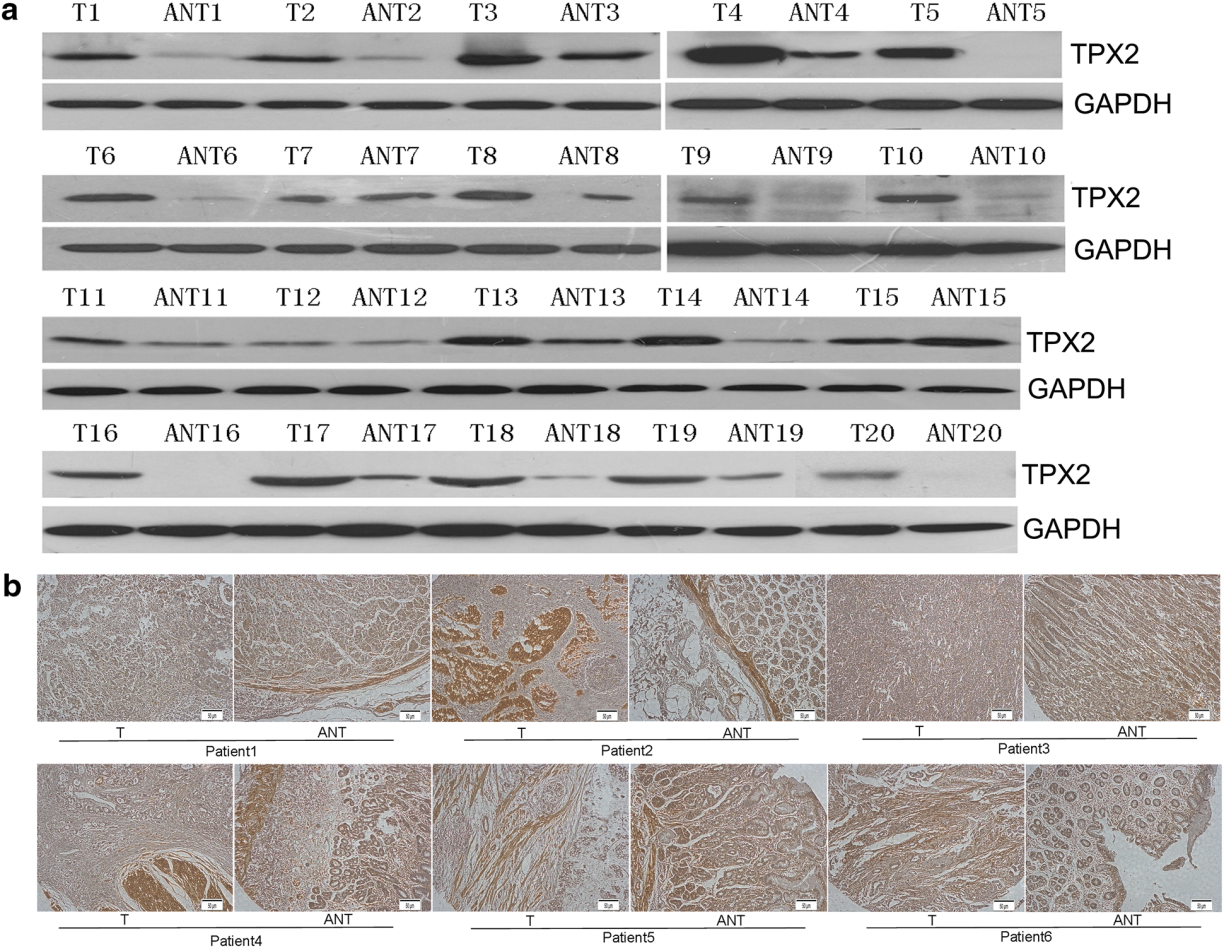
Overexpression of TPX2 protein in human gastric carcinoma tissues. **a** Western blot analyses of TPX2 protein expression in twenty matched pairs of gastric carcinoma (T) and adjacent noncancerous tissues (ANT). GAPDH was used as the loading control. **b** Immunohistochemical assay of TPX2 protein expression in six pairs of matched gastric carcinoma tissues. *T* gastric carcinoma tissues, *ANT* matched adjacent non-tumor gastric tissues (×400)

**Fig. 4:**
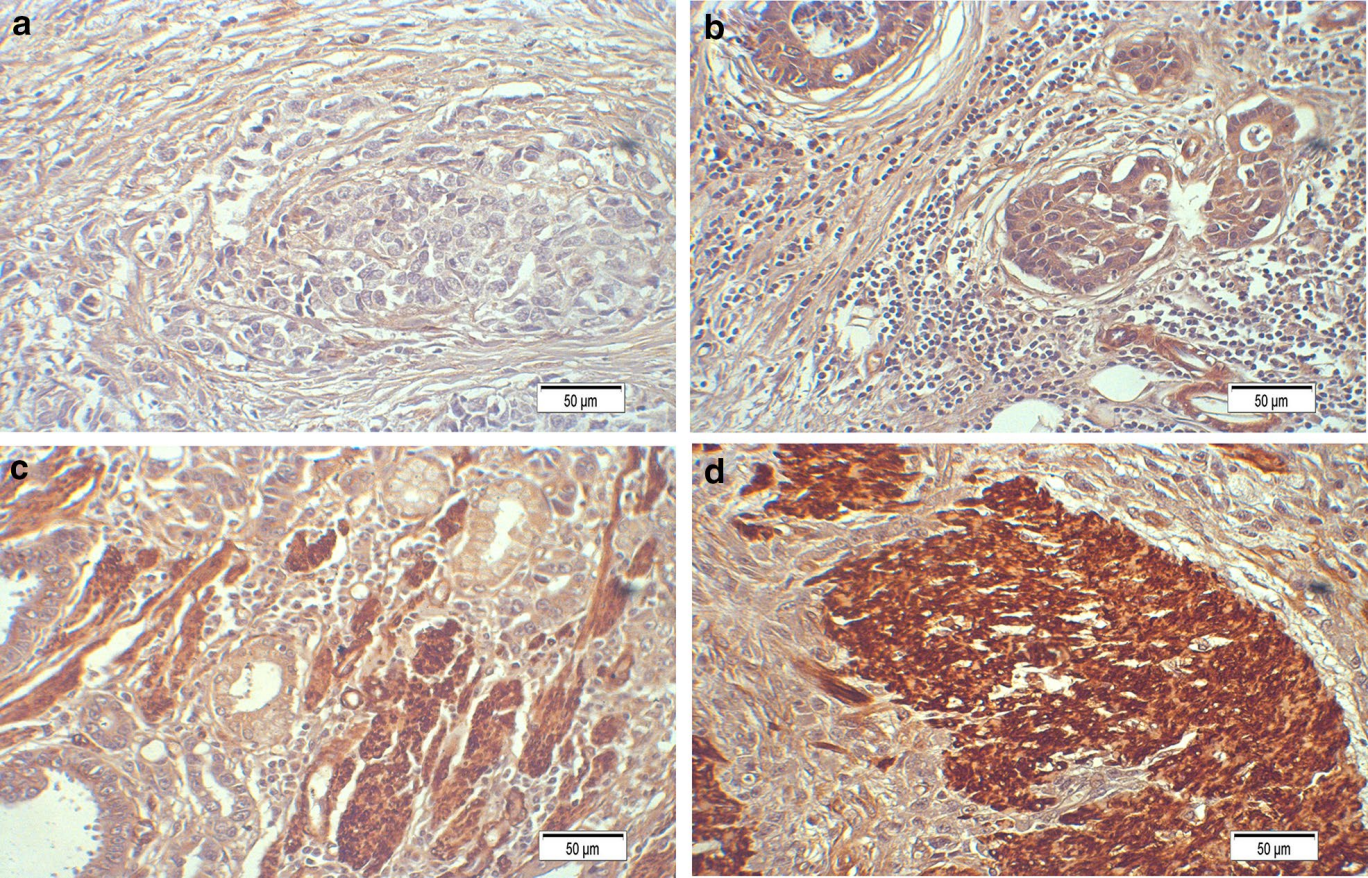
Expression analysis of TPX2 protein by immunohistochemistry. TPX2 expression was mainly localized in the nucleus and cytoplasm of tumor cells. **a** Staining of TPX2 in normal gastric tissues (×400). **b** Low expression of TPX2 in gastric carcinoma tissues (stage II) (×400). **c** Medium expression of TPX2 in gastric carcinoma tissues (stage III) (×400). **d** Higher expression of TPX2 in gastric carcinoma tissues (stage IV) (×400)

**Fig. 5:**
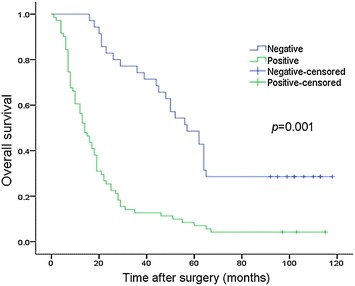
Correlation of TPX2 expression with patient overall survival in gastric carcinoma. Kaplan–Meier curves with univariate analysis (log-rank). OS rates for cases with high TPX2 expression versus those with low TPX2 expression levels in all patients. High TPX2 expression was correlated with an unfavorable prognosis (*P* = 0.001)

To determine whether *TPX2* mRNA expression was different between gastric carcinoma and normal gastric samples, we first queried the Oncomine database (http://www.oncomine.org). In the meta-analysis, *TPX2* expression was significantly higher in the gastric carcinoma tissues than in the corresponding normal tissues (median rank of 187.5, *P* value of 8.51E−8; Fig. 1). To confirm this result, we obtained twenty paired gastric carcinoma and adjacent normal tissues for real-time quantitative polymerase chain reaction (RT-PCR) analysis. As shown in Fig. 2, the expression level of *TPX2* mRNA was significantly higher in tumor tissues (*P* = 0.002). Western blotting analysis revealed that TPX2 protein was differentially increased in 17 of 20 primary human gastric carcinoma tissue specimens compared with the adjacent non-tumor tissues, indicating that TPX2 is up-regulated in gastric carcinoma (Fig. 3a; Additional file 1: Figure S1). However, in the remaining 3 paired tissues, the levels of TPX2 expression were similar, even lower, in tumor tissues than in non-tumor tissues (Fig. 3a; No. 7, 12 and 15). Furthermore, these results were confirmed by immunohistochemistry. The TPX2 positivity rate was 66.98 % (71/106) in tumor tissues. In non-cancerous adjacent tissues, TPX2 protein staining was present in a few cells and was very weak (Figs. 3a, 4). TPX2 was predominantly present in the nucleus and cytoplasm of tumor cells (Fig. 5). These data demonstrated that TPX2 was significantly increased in gastric carcinoma.

### TPX2 is correlated with the clinicopathological features of gastric carcinoma

To obtain a better understanding of the potential roles of TPX2 in gastric carcinoma development and progression, we investigated the relationship between TPX2 expression and other clinicopathological features in 106 paraffin-embedded archived gastric cancer tissues, including 10 stage I tumors, 10 stage II tumors, 84 stage III tumors, and 2 stage IVa tumors. Among 106 samples, high TPX2 protein expression was detected in 71 samples (66.98 %), and weak or no staining was observed in 35 tumor samples (33.02 %; Table 1). As shown in Fig. 3, TPX2 was highly expressed in gastric cancer tissues. By contrast, no signals or only weak signals were detected in adjacent non-cancerous tissues. The subcellular location of TPX2 was mainly in the nucleus and cytoplasm.

**Table 1:** Correlation of TPX2 expression with clinicopathologic features

Characteristics	Total (n = 106)	TPX2		*P* value
		Negative (n = 35)	Positive (n = 71)	
Gender				
Male	67 (63.2 %)	22 (32.8 %)	45 (67.2 %)	0.958
Female	39 (36.8 %)	13 (33.3 %)	26 (66.7 %)	
Age (years)				
≥60	46 (43.4 %)	7 (15.2 %)	39 (84.8 %)	0.001
<60	60 (56.6 %)	28 (46.7 %)	32 (53.3 %)	
T stage				
1	10 (9.4 %)	8 (80 %)	2 (20 %)	0.003
2	10 (9.4 %)	4 (40 %)	6 (60 %)	
3	84 (79.2 %)	22 (26.2 %)	62 (73.8 %)	
4a	2 (1.9 %)	1 (50 %)	1 (50 %)	
N stage				
0	21 (19.8 %)	10 (47.6 %)	11 (35.2 %)	0.120
1	38 (35.8 %)	14 (36.8 %)	24 (63.2 %)	
3	47 (44.3 %)	11 (6.3 %)	36 (3.6 %)	
M stage				
0	99 (93.4 %)	34 (34.3 %)	65 (65.7 %)	0.421
1	7 (6.6 %)	1 (14.3 %)	6 (85.7 %)	
TNM stage				
I	13 (12.3 %)	5 (38.5 %)	8 (61.5 %)	0.144
II	17 (16.0 %)	8 (47.1 %)	9 (52.9 %)	
III	69 (65.1 %)	22 (31.9 %)	47 (68.1 %)	
IV	7 (6.3 %)	0 (0 %)	7 (100 %)	
Tumor size (cm)				
≥5	74 (69.8 %)	24 (32.4 %)	50 (67.6 %)	1.000
<5	32 (30.2 %)	11 (34.4 %)	21 (65.6 %)	

The results of immune-histochemical score were determined by the average score that given by two pathologists, immune-reactivities were scored by the intensity of staining (0, no staining; 1 weak = light yellow; 2 moderate = yellow brown; 3 strong = brown) and the percentage of stained cells (0 no staining; 1, 0–10 %; 2, 11–30 %; 3, 31–70 %; 4, >71 %). By multiplication of both values, a final score ranging between 0 and 12 was obtained. TPX2 over-expression was defined as final score more than zero

We further analyzed the correlation between TPX2 expression and the clinicopathological characteristics of patients. As is summarized in Table 1, TPX2 expression was significantly associated with age (*P* = 0.001) and T stage (*P* = 0.003). However, there was no correlation between TPX2 expression and other clinical features, such as gender, N/M stage, and tumor size (Table 1).

### Univariate and multivariate analyses of the prognosis of gastric carcinoma patients

Univariate and multivariate analyses were carried out using the Cox proportional hazards regression model to investigate the prognostic value of TPX2 expression (Table 2). Age, tumor size, N stage, clinical AJCC stage, and high TPX2 expression predicted a poor prognosis in the univariate analysis (*P* = 0.001, 0.001, 0.000, 0.03 and 0.001, respectively). In the multivariate analysis of the prognostic factors, we confirmed that high TPX2 expression was an independent prognostic factor (95 % CI 2.626–7.198; *P* = 0.001), and age, tumor size, and N stage were also independent and significant prognostic factors for OS (*P* = 0.046, 0.005 and 0.000).

**Table 2:** Cox-regression analysis of various prognostic parameters in patients for all patients

Factor	Univariate		Multivariate	
	HR (95 % CI)	*P* value	HR (95 % CI)	*P* value
Sex				
Male	Reference			
Female	0.883 (0.581–1.340)	0.558	–	–
Age				
≥60	Reference			
<60	2.077 (1.375–3.139)	0.001	1.561 (1.009–2.417)	0.046
Tumor size (cm)				
<5	Reference			
≥5	2.277 (1.410–3.678)	0.001	2.091 (1.249–3.502)	0.005
TPX2 expression				
Negative	Reference			
Positive	3.703 (2.303–5.956)	0.001	4.708 (2.800–7.917)	0
AJCC tumor stage				
I	Reference			
II	1.971 (0.840–4.624)	0.119	–	–
III	2.268 (1.081–4.756)	0.030	–	–
IV	2.666 (0.963–7.382)	0.059	–	–
N stage				
0	Reference			0
1	4.022 (1.955–8.274)	0.000	3.122 (1.474–6.611)	0.003
3	7.015 (3.421–14.386)	0.000	5.858 (2.731–12.566)	0
Distant metastasis				
No	Reference			
Yes	0.855 (0.346–2.110)	0.733	–	–
Infiltration				
No	Reference			
Yes	2.023 (0.639–6.402)	0.231	–	–

### Expression of TPX2 in gastric carcinoma patients correlates with a worse overall survival

After a median follow-up of 101 months (range 1–118 months), 89 (83.1 %) patients died. The estimated 5-year OS was 21 % (95 % CI 17.122–24.878). Survival analysis showed a clear negative correlation between the TPX2 protein expression level and OS of patients with gastric cancer (*P* = 0.001; Fig. 5). Patients with negative TPX2 expression had an estimated 5-year OS rate of 57 % (95 % CI 43.091–70.909), whereas patients with positive TPX2 expression had a 5-year OS rate of 14 % (95 % CI 10.333–17.667, *P* = 0.001; Fig. 5). These results indicated that TPX2 expression may be an independent prognostic marker for OS in gastric carcinoma patients.

## Discussion

Gastric carcinoma is one of the main cancer-related causes of death worldwide [1, 12]. The curative treatment of gastric carcinoma consists of tumor resection and lymphadenectomy [13]. However, surgical treatment alone is associated with high recurrence rates [14]. Adjuvant treatment strategies have demonstrated controversial results [15]. Despite improvement in early detection and treatments, the outcome of advanced gastric carcinoma remains unsatisfactory due to the poor understanding of the intricate pathogenesis of gastric carcinoma. Gastric carcinoma is a multifactorial and multistep disease [16] that involves activation of oncogenes and inactivation of tumor suppressor genes [17]. A multimodal approach has been demonstrated to significantly increase OS, and it can be offered in the form of perioperative chemotherapy, adjuvant chemoradiotherapy, or adjuvant chemotherapy [18]. Recently, numerous studies have investigated the molecular basis of gastric carcinoma [19], involving the alteration of pathogenesis, as well as invasion and metastasis [20, 21]. With the development of modern technologies, various novel biomarkers have been identified that appear to possess diagnostic and prognostic value [22, 23]. Identifying useful biomarkers for the early diagnosis and prognosis of gastric carcinoma is an urgent need.

TPX2 has been identified as a mitotic regulator involved in the formation of the mitotic spindle [24]. This protein, as well as its partner, Aurora-A, is frequently overexpressed in many tumor types [25], and its deregulation may participate not only in chromosomal numerical aberrations but also in other forms of genomic instability in cancer cells [26]. Overexpressed in cancers, TPX2 is regarded as a novel candidate target for the diagnosis and prognosis of malignancies [11]. During interphase, TPX2 resides preferentially in the nucleus, where its function has remained elusive until recently [11]. The latest finding that TPX2 plays a role in amplification of the DNA damage response [27], combined with the characterization of TPX2 knockout mice [26], provides a new perspective in understanding the signaling roles of TPX2 as they relate to cancer therapies. Additionally, it was found that the expression of TPX2 was associated with tumor grade, stage, lymph node metastasis, and tumor size pathological type [28].

Based on previous studies, we investigated the expression of TPX2 in gastric carcinoma and its prognostic value in predicting OS following curative resection in the present study. Our results clearly showed that TPX2 was up-regulated at both the mRNA and protein levels in twenty gastric carcinoma tissues compared with matched non-tumor gastric tissues. In addition, IHC analysis showed that TPX2 was overexpressed in 78 of 106 (73.58 %) gastric carcinoma specimens. The difference in the expression of TPX2 between gastric carcinoma and non-tumor tissues indicates that TPX2 may be a key regulator in tumorigenesis and the prognosis of gastric carcinoma.

We further analyzed the relationship between the expression of TPX2 and the clinical characteristics of patients with gastric carcinoma. There was a significant correlation of TPX2 expression with age (*P* = 0.001) and T stage (*P* = 0.003). However, there was no correlation of TPX2 expression with other clinical features, such as gender, N/M stage, and tumor size, in contrast to the findings of other studies. This discrepancy may be due to the differences in ethnicity and sample size between the studies.

Multivariate analysis revealed that TPX2 expression, age, tumor size, and N stage may be independent prognostic indicators of OS in gastric carcinoma patients. This finding indicates the possibility of using high expression levels of TPX2 as a predictor of prognosis and survival.

However, sex, AJCC tumor stage, distant metastasis, and infiltration were not independent prognostic indicators of OS in gastric carcinoma patients. It is unclear why AJCC clinical stage was not found to be an independent prognostic indicator of OS in gastric carcinoma patients. A possible reason is the small sample size in this study. Thus, we plan to collect more patients at different clinical centers and perform an in-depth investigation of the mechanism of TPX2 action in our future research.

To our knowledge, this is the first report on TPX2 in gastric carcinoma, which was found to be an independent predictor of OS in gastric carcinoma patients. Further studies are warranted to elucidate the molecular mechanism of TPX2 in the development and progression of gastric carcinoma.

## Additional file

10.1186/s12935-016-0357-7 Western blot analyses show the molecular weight of TPX2 was about 85 KD. T, gastric carcinoma tissues. ANT, adjacent noncancerous tissues.

## Abbreviations

TPX2: xenopus kinesin-like protein 2
RT-PCR: real-time quantitative polymerase chain reaction
TMA: tissue microarray
OS: overall survival
HR: hazard ratio
CI: confidence interval
GAPDH: glyceraldehyde-3-phosphate dehydrogenase
SDS-PAGE: sodium dodecyl sulfate-polyacrylamide gel electrophoresis
PVDF: polyvinylidene fluoride
HE: hematoxylin and eosin
IHC: immunohistochemistry
DAB: 3, 3′-diaminobenzidine
AJCC: American Joint Committee on Cancer
UICC: Union for International Cancer Control

## References

[1] Ferro A, Peleteiro B, Malvezzi M, Bosetti C, Bertuccio P, Levi F, Negri E, La Vecchia C, Lunet N (2014). Worldwide trends in gastric cancer mortality (1980–2011), with predictions to 2015, and incidence by subtype. Eur J Cancer.

[2] Bollschweiler E, Berlth F, Baltin C, Monig S, Holscher AH (2014). Treatment of early gastric cancer in the Western World. World J Gastroenterol.

[3] Lan H, Zhu N, Lan Y, Jin K, Teng L (2015). Laparoscopic gastrectomy for gastric cancer in China: an overview. Hepatogastroenterology.

[4] Zhu WG, Xua DF, Pu J, Zong CD, Li T, Tao GZ, Ji FZ, Zhou XL, Han JH, Wang CS (2012). A randomized, controlled, multicenter study comparing intensity-modulated radiotherapy plus concurrent chemotherapy with chemotherapy alone in gastric cancer patients with D2 resection. Radiother Oncol.

[5] Hingorani M, Dixit S, Johnson M, Plested V, Alty K, Colley P, Beavis AW, Roy R, Maraveyas A (2015). Palliative radiotherapy in the presence of well-controlled metastatic disease after initial chemotherapy may prolong survival in patients with metastatic esophageal and gastric cancer. Cancer Res Treat.

[6] Chen J, Hong D, Zhai Y, Shen P (2015). Meta-analysis of associations between neutrophil-to-lymphocyte ratio and prognosis of gastric cancer. World J Surg Oncol.

[7] Xu X, Chang X, Li Z, Wang J, Deng P, Zhu X, Liu J, Zhang C, Chen S, Dai D (2015). Aberrant SOX11 promoter methylation is associated with poor prognosis in gastric cancer. Cell Oncol (Dordr).

[8] Goshima G (2011). Identification of a TPX2-like microtubule-associated protein in *Drosophila*. PLoS One.

[9] Petry S, Groen AC, Ishihara K, Mitchison TJ, Vale RD (2013). Branching microtubule nucleation in Xenopus egg extracts mediated by augmin and TPX2. Cell.

[10] Zorba A, Buosi V, Kutter S, Kern N, Pontiggia F, Cho YJ, Kern D (2014). Molecular mechanism of Aurora A kinase autophosphorylation and its allosteric activation by TPX2. Elife.

[11] Neumayer G, Belzil C, Gruss OJ, Nguyen MD (2014). TPX2: of spindle assembly, DNA damage response, and cancer. Cell Mol Life Sci.

[12] Peleteiro B, Castro C, Morais S, Ferro A, Lunet N (2015). Worldwide burden of gastric cancer attributable to tobacco smoking in 2012 and predictions for 2020. Dig Dis Sci.

[13] Merrett ND (2014). Multimodality treatment of potentially curative gastric cancer: geographical variations and future prospects. World J Gastroenterol.

[14] Biondi A, Cananzi FC, Persiani R, Papa V, Degiuli M, Doglietto GB, D’Ugo D (2012). The road to curative surgery in gastric cancer treatment: a different path in the elderly?. J Am Coll Surg.

[15] Saito M, Kiyozaki H, Takata O, Suzuki K, Rikiyama T (2014). Treatment of stage IV gastric cancer with induction chemotherapy using S-1 and cisplatin followed by curative resection in selected patients. World J Surg Oncol.

[16] Zabaleta J (2012). Multifactorial etiology of gastric cancer. Methods Mol Biol.

[17] Hosoda F, Arai Y, Okada N, Shimizu H, Miyamoto M, Kitagawa N, Katai H, Taniguchi H, Yanagihara K, Imoto I (2015). Integrated genomic and functional analyses reveal glyoxalase I as a novel metabolic oncogene in human gastric cancer. Oncogene.

[18] Mattiucci GC, Valentini C, D’Agostino GR, Augurio A, Capirci C, De Paoli A, Genovesi D, Huscher A, Iannone T, Pani G (2015). Adjuvant chemoradiotherapy in gastric cancer: a pooled analysis of the AIRO gastrointestinal group experience. Tumori.

[19] Stiekema J, Trip AK, Jansen EP, Boot H, Cats A, Ponz OB, Verheij M, van Sandick JW (2014). The prognostic significance of an R1 resection in gastric cancer patients treated with adjuvant chemoradiotherapy. Ann Surg Oncol.

[20] Zhang KC, Xi HQ, Cui JX, Shen WS, Li JY, Wei B, Chen L (2015). Hemolysis-free plasma miR-214 as novel biomarker of gastric cancer and is correlated with distant metastasis. Am J Cancer Res.

[21] Yamashita K, Kuno A, Matsuda A, Ikehata Y, Katada N, Hirabayashi J, Narimatsu H, Watanabe M (2015). Lectin microarray technology identifies specific lectins related to lymph node metastasis of advanced gastric cancer. Gastric Cancer.

[22] Wu HH, Lin WC, Tsai KW (2014). Advances in molecular biomarkers for gastric cancer: miRNAs as emerging novel cancer markers. Expert Rev Mol Med.

[23] Wang H, Wang L, Wu Z, Sun R, Jin H, Ma J, Liu L, Ling R, Yi J, Bian J (2014). Three dysregulated microRNAs in serum as novel biomarkers for gastric cancer screening. Med Oncol.

[24] Wei P, Zhang N, Xu Y, Li X, Shi D, Wang Y, Li D, Cai S (2013). TPX2 is a novel prognostic marker for the growth and metastasis of colon cancer. J Transl Med.

[25] Hsu PK, Chen HY, Yeh YC, Yen CC, Wu YC, Hsu CP, Hsu WH, Chou TY (2014). TPX2 expression is associated with cell proliferation and patient outcome in esophageal squamous cell carcinoma. J Gastroenterol.

[26] Liu HC, Zhang Y, Wang XL, Qin WS, Liu YH, Zhang L, Zhu CL (2013). Upregulation of the TPX2 gene is associated with enhanced tumor malignance of esophageal squamous cell carcinoma. Biomed Pharmacother.

[27] Neumayer G, Nguyen MD (2014). TPX2 impacts acetylation of histone H4 at lysine 16: implications for DNA damage response. PLoS One.

[28] Aguirre-Portoles C, Bird AW, Hyman A, Canamero M, Perez de Castro I, Malumbres M (2012). Tpx2 controls spindle integrity, genome stability, and tumor development. Cancer Res.

