# Supplementary figures and images for: Expression of microtubule-associated protein TPX2 in human gastric carcinoma and its prognostic significance
Source: Cancer Cell Int. 2016 Oct 10;16:79. doi: 10.1186/s12935-016-0357-7 (PMC5057506; doi:10.1186/s12935-016-0357-7)

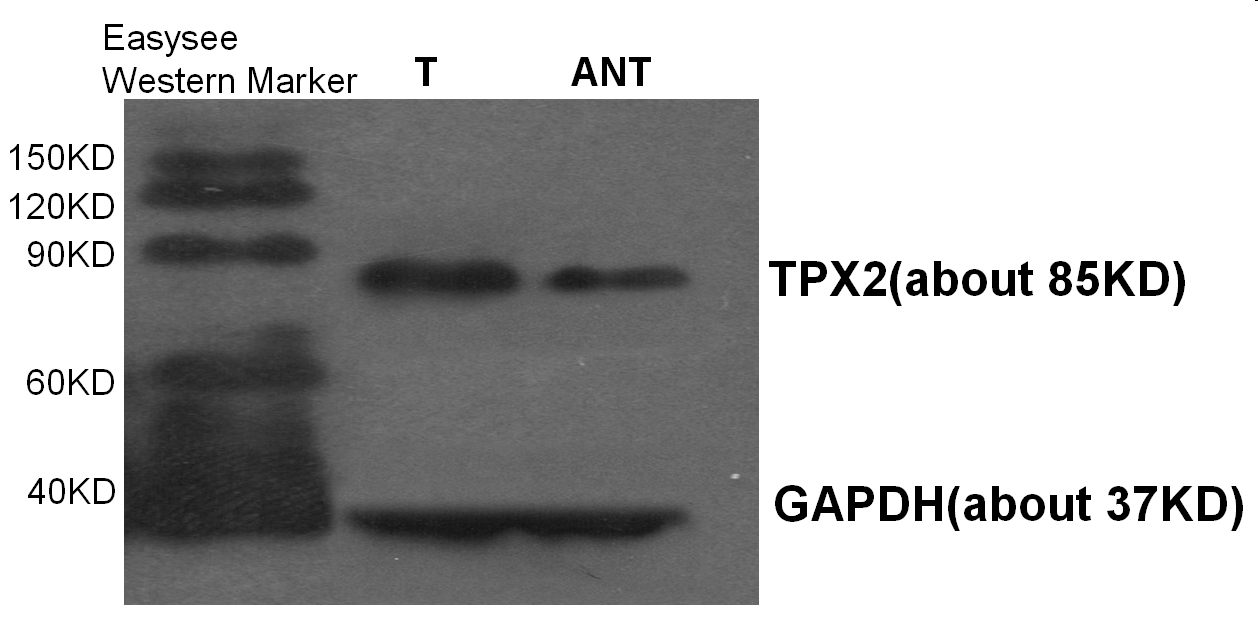

Supplement: Supplementary file 1 — 10.1186/s12935-016-0357-7 Western blot analyses show the molecular weight of TPX2 was about 85 KD. T, gastric carcinoma tissues. ANT, adjacent noncancerous tissues. [file 12935_2016_357_MOESM1_ESM.tif]
